# The Power of Three: Coral Reefs, Seagrasses and Mangroves Protect Coastal Regions and Increase Their Resilience

**DOI:** 10.1371/journal.pone.0158094

**Published:** 2016-07-13

**Authors:** Greg Guannel, Katie Arkema, Peter Ruggiero, Gregory Verutes

**Affiliations:** 1 The Natural Capital Project, Stanford University, Stanford, CA, United States of America; 2 The Natural Capital Project, Stanford University, c/o School of Environmental and Forest Sciences, University of Washington, Seattle, WA, United States of America; 3 College of Earth, Ocean, and Atmospheric Sciences, Oregon State University, Corvallis, OR, United States of America; Università di Genova, ITALY

## Abstract

Natural habitats have the ability to protect coastal communities against the impacts of waves and storms, yet it is unclear how different habitats complement each other to reduce those impacts. Here, we investigate the individual and combined coastal protection services supplied by live corals on reefs, seagrass meadows, and mangrove forests during both non-storm and storm conditions, and under present and future sea-level conditions. Using idealized profiles of fringing and barrier reefs, we quantify the services supplied by these habitats using various metrics of inundation and erosion. We find that, together, live corals, seagrasses, and mangroves supply more protection services than any individual habitat or any combination of two habitats. Specifically, we find that, while mangroves are the most effective at protecting the coast under non-storm and storm conditions, live corals and seagrasses also moderate the impact of waves and storms, thereby further reducing the vulnerability of coastal regions. Also, in addition to structural differences, the amount of service supplied by habitats in our analysis is highly dependent on the geomorphic setting, habitat location and forcing conditions: live corals in the fringing reef profile supply more protection services than seagrasses; seagrasses in the barrier reef profile supply more protection services than live corals; and seagrasses, in our simulations, can even compensate for the long-term degradation of the barrier reef. Results of this study demonstrate the importance of taking integrated and place-based approaches when quantifying and managing for the coastal protection services supplied by ecosystems.

## Introduction

Increasingly, coastal and marine ecosystems are perceived, promoted and used as alternatives to traditional coastal protection structures [1–4]. The hope is that, through conservation and restoration efforts, these ecosystems will shield communities from the impacts of coastal hazards [5–9], while continuing to deliver services that increase the well-being of residents, and improve the ecological health of coastal regions [10–13]. Accordingly, governments are promoting approaches to coastal hazard planning that account for the supply and delivery of multiple benefits from natural systems [14–16]. However, by and large, most proposed nature-based approaches for coastal protection rely on maximizing protection benefits supplied by specific habitats on a seascape (e.g., a wetland, a coral reef, etc.) [4–6,8], using a specific set of forcing conditions that are usually storm conditions [1,6,7,17].

The typical approach of using a single habitat to only protect coastal regions against specific forcing conditions merely treats natural systems as alternatives to traditional mono-functional hard coastal structures, and it underutilizes the potential of all the habitats present on the entire seascape. Indeed, this approach overlooks the fact that natural systems can help protect coasts from a host of hazards that occur under different forcing conditions. For example, while coral reefs and wetlands can reduce the impacts of tsunamis, storm surge and storm waves [2,18,19], they can also moderate wind-waves and swells, and thus reduce chronic shoreline erosion, promote shoreline accretion, or create conditions conducive to wetlands reproduction [8,20–22]. In addition, a single-habitat approach to coastal protection overlooks the possibility that structurally different natural systems on a seascape can together supply higher levels of protection services by progressively moderating the impacts of hydrodynamic processes, as shown heuristically by Refs. [23–25]. In other words, typical nature-based protection schemes, which have traditionally focused on a single habitat rather than a whole system approach [2,3,21], ignore the temporal and cumulative moderating effects of all habitats on a seascape. Thus, comprehensive methods to design integrated management approaches that maximize the coastal protection services delivered by entire seascapes under various climatic conditions are lacking.

**Fig 1:**
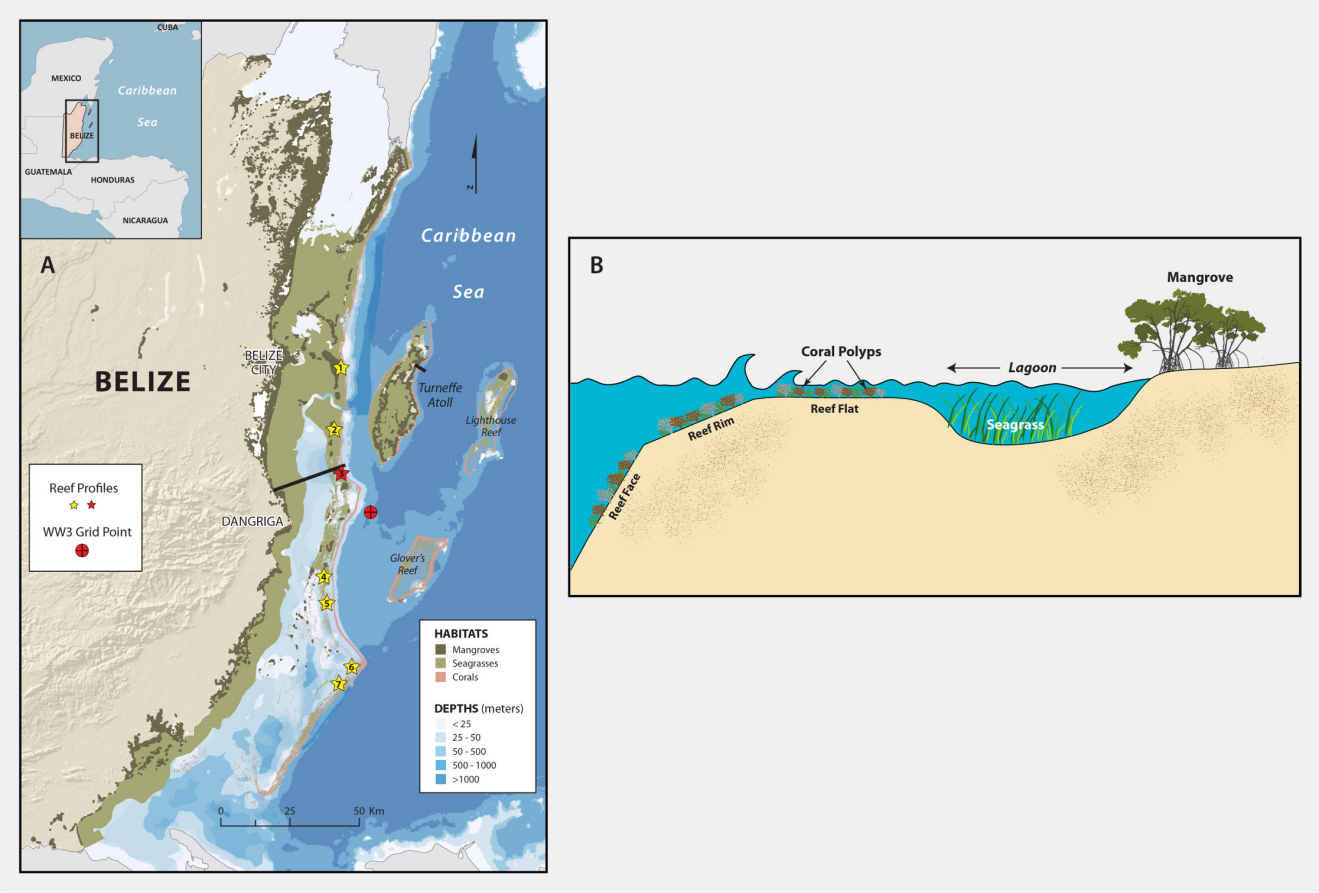
Typical tropical seascapes found in Belize. A) Map of Belize showing the location of coral reefs, seagrass meadows and mangrove forests. Yellow stars indicate the location of profiles measured by Burke [26]; the red star indicates the “Colson” profile used herein. The thin black lines represent the locations of the two profiles (Dangriga and Turneffe Atoll) approximated in this analysis. B) Typical schematic of the Belizean seascape.

**Fig 2:**
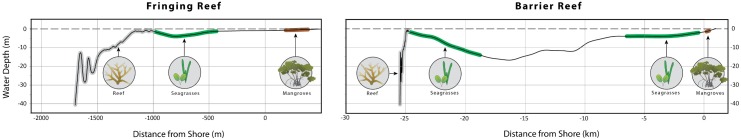
Idealized bathymetric profiles of fringing and barrier reefs.

Here, using the Caribbean country of Belize as a characteristic setting (Fig 1), we quantify the coastal protection services supplied by two 1-Dimensional (1-D) idealized seascapes: one bounded offshore by a barrier reef and the other by a fringing reef (Figs 1 & 2; Table 1), with seagrass meadows present in the lagoons, and mangrove forests growing at the coastlines. We investigate the relative importance of the three different habitats at protecting the coast under non-storm conditions and during a typical hurricane, using 8 scenarios of habitat presence/absence (e.g., no habitats; live corals present only; live corals and seagrass present, but no mangroves; live corals, seagrass and mangroves present etc.; Table 1). In particular, we investigate the relative importance of live coral on reefs by comparing the level of protection offered by reefs covered with live corals compared with reefs covered with dead corals. We also evaluate the importance of live corals on reefs by conducting the analyses under two sea-level scenarios: a present day sea-level and a future 1 m sea-level rise (SLR) scenario, assuming that reefs covered with live corals will accrete as sea-level rises. For all simulations, we use a variety of metrics that can be converted to avoided damages to coastal property and avoided loss of land. These include reduction in wave height, bed shear stress, inundation level and volume of mud bed scour.

**Table 1:** Categorization of habitat and forcing scenarios for two tropical seascapes (fringing reef and barrier reef).

Time Horizon	Present	Future
**Forcing Conditions**	(a) Non-storm conditions; (b) Storm (hurricane) conditions	
**Sea-Level Increase**	0 m	+1 m
**Coral Reef Scenarios**	(a) Live corals on reef surface (*C*_*f*_ = 0.2)*; (b) Dead corals on reef surface (*C*_*f*_ = 0.1).	(a) Live corals on reef surface (*C*_*f*_ = 0.2), reef accretes with SLR^†^; (b) No corals on reef surface (*C*_*f*_ = 0.05), reef does not accrete with SLR
**Seagrasses Scenarios**	Meadow present/absent^‡^; Drag coefficient *C*_*d*_ ∼ 0.1 during non-storm conditions, and *C*_*d*_ ∼ 0.05 during storm conditions	
**Mangroves Scenarios**	Forest present/absent; Drag coefficient *C*_*d*_ = 1 during non-storm and storm conditions	

*C_*f*_ is a friction coefficient; see Eq 6.

^†^SLR: Sea-Level Rise

^‡^Differences in seagrass coverage between the reef profiles reflect conditions in Belize; see Figs 1 & 2.

## Methods

### Study System

Tropical seascapes generally have 3 distinct habitat types: coral reefs, seagrass meadows and mangrove forests. The coral reef may be a barrier reef, a fringing reef or an atoll, and is covered by coral colonies. Seagrass meadows generally grow shoreward of the reef, and they cover as much of the seabed as light and water quality conditions allow. Mangrove forests occupy the nearshore intertidal and coastal regions, extending from mean lower low water (MLLW) to well landward of the mean higher high water (MHHW) mark.

We quantify the difference in coastal protection services supplied by habitats living in two idealized tropical 1-D seascapes: one bounded offshore by a barrier reef and the other by a fringing reef (Figs 1 & 2; Table 1). At both sites we use the “Colson” reef profile measured by Burke [26], where, during present day sea-level conditions, the reef flat is 1 m below mean sea level (MSL, the reference for all depths or elevations reported herein), with some regions shallower than 20 cm [26]. Shoreward of the reef, we re-created the lagoon profiles based on observed and measured characteristics of the Belizean seascape [26,27] (Fig 2): the fringing reef lagoon is 0.3 km long with an average depth of 3 m; the barrier reef lagoon is 25 km long with a maximum depth of 17 m (Fig 2). We complete these transects by adding to both lagoon profiles a typical 1V:1000H mud flat from -0.5 m MSL to MHW (approximately +0.3 m MSL [28]) followed by a typical 1V:500H mangrove forest mud floor [29,30].

Existing seagrass coverage patterns in Belize (Fig 1) show that regions deeper than 1 m and shallower than 13 meters are fully or partially covered with the commonly found Turtle Grass (*Thalassia testudinum*) meadows [31]. Here, the barrier reef lagoon has two, roughly 6 km wide seagrass meadows (Figs 1 & 2). In contrast, most of the fringing reef lagoon is covered with seagrass. In both profiles, we assume that seagrasses have a typical stem diameter of *d*_*v*_ = 0.1 cm, height of *h*_*v*_ = 30 cm [32,33], and stem density averages *N*_*v*_ = 250 stems/m^2^ [34]. At the shoreline, a dense red mangrove forest (*Rhizophora mangle*) [35] extends 200 m landward from MLLW (MLLW is approximately at -0.3 m in the Caribbean [28]). Mangrove roots (subscript *r*) and trunks (subscript *t*) have typical diameters of *d*_*vr*_ = 2 and *d*_*vt*_ = 20 cm, heights of *h*_*vr*_ = 0.5 and *h*_*vt*_ = 2 m, and densities of *N*_*vr*_ = 90 and *N*_*vt*_ = 90 units/m^2^ [30,36].

To quantify the long-term importance of live coral cover, we create two future reef profiles for both the barrier and the fringing reef. These two reef profiles represent future conditions where sea-level has risen by 1 m [37] (Table 1). For the first future profile (“live reef” profile), the reef is initially covered with live corals and accretes vertically as sea-level rises. (To “keep up” with sea-level rise, the reef must accrete at a rate greater than 10 mm/yr, which is relatively high for the Caribbean [38].) For the second future profile (“dead reef” profile), the reef is initially covered with dead corals, and thus becomes flatter and smoother with time because of wave action and bio-erosion [39,40]; this reef does not accrete, exhibits little three-dimensional structure, and can be treated as flat bedrock [41,42]. Lastly, we deepen the lagoons by 1 m, and extend the seagrass meadows so they grow within the same depth ranges as in the present day scenario. At the coast, we assume the mud flat and mangrove forest migrate landward by 500 m to keep pace with sea-level rise (we assumed a 1V:500H mangrove floor slope).

The 1-D profiles described above represent typical, yet idealized, reef profiles found in tropical seascapes. The physical characteristics of the seagrass meadows and mangrove trees are based on average values found in the literature. Consequently, results presented herein are an indication of the services supplied by different habitats on the synthetic seascapes, and are not specific estimates of the services supplied by habitats in particular locations in Belize.

### Forcing Conditions

We quantify the protective role of coral reefs, seagrass meadows and mangroves under both non-storm and storm conditions (Table 1). During non-storm conditions, the wave climate consists of locally generated wind waves and of swells originating from distant storms. We created a record for non-storm forcing conditions by post-processing 9 years (2005 to 2013) of WaveWatch III (WW3) [43] data offshore of the Belizean barrier reef (Fig 1), where we excluded all wave data between June and October, the hurricane season, and removed all data 10 hours before and after any occurences of wind speed faster than 17 m/s, the minimum speed during tropical storms [44]. As a result, the dataset used in the modeling effort was reduced from more than 24,000 records to just over 14,000 (S1 Fig). Waves statistics are presented in Table 2.

**Table 2:** Non-Storm Wave Statistics Offshore of Belize.

	Wave Height [m] (Wave Period [s])*	Wave Power [kW/m] (Wave Height [m] and Period [s])^†^
Mean	1.4 (6.8)	7.7 (1.5, 7.1)
Maximum	3.8 (9.1)	66 (3.8, 9.1)

*Average wave period associated with repeated values of the mean and maximum wave height ±0.2 m.

^†^Average wave height and period associated with repeated values of the mean and maximum wave power ± 0.2 kW/m.

To capture the protection services delivered by natural habitats under storm conditions we created a synthetic hurricane wind field [45] with regionally appropriate values of central pressure of 980 mb, radius of maximum winds *R*_*max*_ of 47 km [46], and forward speed *V*_*fm*_ of 8.5 km/hr [47]. A storm with these representative characteristics generates a maximum sustained wind speed *W*_*max*_ of 101 km/hr and a storm surge height on the mainland of approximately 1 m [44], which are typical values for this region during hurricanes [48].

In addition to storm-induced surges, hurricanes generate high waves that increase the extent of coastal erosion and inundation. Following Young [49], our synthetic hurricane generates offshore of the two reef profiles a deep water significant wave height *H*_*o*_ = 9.5 m, with a peak period *T*_*p*_ = 13.1 s.

### Numerical Model for the Evolution of Waves and Storm Surge

We quantify the supply of protection services provided by the habitats on the two seascapes by modeling the evolution of waves and storm surge over the 1-D bathymetric profiles, neglecting two-dimensional and non-linear processes [50,51]. Thus, we only consider cross-shore processes and ignore planform evolution processes. We also assume that the reefs’ side channels are sufficiently wide so that the lagoons are well flushed, and wave setup at the shoreward edge of the reef and infragravity wave energy in the lagoons are negligible [52–54]. Further, the hurricane moves at a constant speed, on a path perpendicular to the shoreline, and it does not uproot or damage vegetation.

#### Storm Surge Generation

We compute the surge generated by the synthetic hurricane by linearly adding the barometric surge and wind setup produced by the storm [55]

$$S=S_{B}+S_{w}$$ (1)

The barometric surge, *S*_*B*_, is computed as *S*_*B*_ = 0.0104(*P*_∞_ − *P*_*o*_) [55], where *P*_∞_ is the normal atmospheric pressure, and *P*_*o*_ the pressure at the center of the hurricane. The wind setup, *S*_*w*_, is computed as [55]:

$$\{\begin{array}{l} \;g\left(h+S_{w}\right)\frac{\partial S_{w}}{\partial x}=\left(h+S_{w}\right)f_{C}V+\tau_{wx}\\ \frac{\partial V}{\partial t}=\frac{\tau_{wy}-\tau_{by}}{\rho \left(h+S_{w}\right)} \end{array}$$ (2)

where *ρ* is the water density, *g* the constant of gravity, *f*_*C*_ the Coriolis parameter, *h* is the water depth relative to MSL, and *x* is the cross-shore distance from the offshore-most point of each profile (Fig 2). In addition, *V* is the wind-induced longshore current, and *τ*_*wy*_ and *τ*_*by*_ are surface wind and bed shear stresses [55].

At the coast, we compute inundation extent with a bathtub approach, where any land area below the surge elevation at the shoreline is flooded. We also assume that mangroves reduce storm surge linearly at the rate of 40 cm/km [18], but that reefs and seagrasses have a negligible effect.

#### Wave Evolution

For both storm and non-storm scenarios, we model the evolution of the wave field over the reef profiles (Table 1) by solving the well-established wave evolution equation [56,57]:

$$\frac{\partial E_{w}C_{g}}{\partial x}=S_{Wind}-D_{b}-D_{f}-D_{v}$$ (3)

where *x* represents the distance from the most offshore point along the profile, *E*_*w*_ is the wave energy density, *C*_*g*_ is the wave group velocity, and *S*_*Wind*_ is an energy source term that allows waves in the lagoons to re-generate under the action of hurricane winds [58]. This term can be expressed as [56]:

$$S_{Wind}=A_{1}\rho g{\left(\frac{W}{H_{o}}\right)}^{2}f\left(H_{o},T_{p},W,h_{t}\right)$$ (4)

where *A*_1_ is a constant, *W* the offshore sustained wind speed, and *h*_*t*_ the local water depth. The exact formulation of the function *f* is derived and discussed in the technical documentation of FEMA’s 1D wave model *Whafis* [56]. We compute *S*_*Wind*_ over open water areas using the hurricane maximum sustained wind speed [59]. At the coast, we assume that mangrove canopies block the winds, and *S*_*Wind*_ becomes zero [56].

Waves gain energy from winds, but they also dissipate their energy via breaking, as expressed by the term *D*_*b*_ in Eq (3) [60,61]:

$$D_{b}=\frac{1}{8\pi }\rho g\sigma Q_{b}b\;H_{m}^{2}$$ (5)

where *σ* is the wave radial frequency, and *Q*_*b*_ is the breaking probability of the waves computed by solving [60]: (1−*Q*_*b*_)/ln *Q*_*b*_ = −(*H* / *H*_*m*_)^2^, where *H*_*m*_ = *γh* is the maximum wave height, with *γ* a breaker index, and *b* is a breaking coefficient. Filipot et al. [61] suggest values of *γ* = 0.69 and *b* = 1.09 for waves travelling over reefs faces, rims and flats. In lagoons and mangrove forests, we use *γ* = 0.73 and *b* = 1 [57,60].

In addition to breaking, waves further dissipate their energy via bottom friction, term *D*_*f*_ in Eq (3) [62]:

$$D_{f}=\frac{C_{f}}{16\sqrt{\pi }}{\left(\frac{\sigma }{\sinh kh}\right)}^{3}H^{3}$$ (6)

where *C*_*f*_ is a friction coefficient corresponding to different bottom roughness values. Corals supply protection services by increasing the roughness of the reefs [42,63]. Adapting the approach of Sheppard et al.[42], we use *C*_*f*_ = 0.2 for live reefs in Eq (6) and *C*_*f*_ = 0.1 for dead reefs that still have coral skeletons in place or scattered around (Table 1). For a long-deceased reef, where wave action and bio-erosion smooth the reef surface [38,40,64], we use *C*_*f*_ = 0.05, a value appropriate for bedrock [41] (we will refer to such a smooth reef as “bare reef”).

Finally, seagrasses and mangroves dissipate wave energy by exerting a drag force on the water column [65], term *D*_*v*_ in Eq (3), expressed as [57]:

$$D_{v}=\frac{1}{2\sqrt{\pi }}\rho {\left(\frac{kg}{2\sigma }\right)}^{3}\frac{\left[D_{r}+D_{t}\right]}{3k\cosh^{3}kh}H^{3}$$ (7)

where *D*_*r*_ and *D*_*t*_ represent dissipation due to the roots and trunk respectively, and their complete expressions are provided in Guannel et al. [51]. These dissipation terms are a function of the diameter *d*_*v*_, density *N*_*v*_, height *h*_*v*_, and drag coefficient *C*_*d*_ associated with each part of the plants. For seagrasses, where roots are buried and there is no canopy, *D*_*r*_ = 0. This expression has been extensively validated against multiple datasets [51,66].

We compute the amount of wave energy dissipated by vegetation at each cross-sectional grid point using the plants’ physical characteristics described in Section 2.1. We also use a drag coefficient of *C*_*dr*,*t*_ = 1 for mangroves [67] since roots and trunks are not flexible. Seagrass, on the other hand, is highly flexible. As observed by Zeller et al. [68], seagrass stems sway under the action of moderately energetic waves and are fully bent under the action of larger waves. Hence the drag coefficient associated with seagrasses varies widely under different hydrodynamic conditions (see, e.g, Refs [51,66] for more details). Accordingly, during non-storm conditions, we compute *C*_*d*_ for Turtle Grass following Bradley et al. [69], where *C*_*d*_ ∼ 0.1. During hurricanes, where the Reynolds number becomes quite large, we lower the drag coefficient to *C*_*d*_ = 0.005, following observations by Zeller et al. [68].

Application of Eq (3) to various linear bathymetry profiles and with different wind speeds produced waves comparable to outputs from the model *SWAN* [70] with Generation 1 or 2 wind-wave formulations. This equation has also been shown to capture the bulk properties of non-storm waves travelling over reefs and in lagoons [71,72], and has been extensively validated in the presence of vegetation, as summarized in Refs. [66] and [51].

#### Protection Metrics

**Fig 3:**
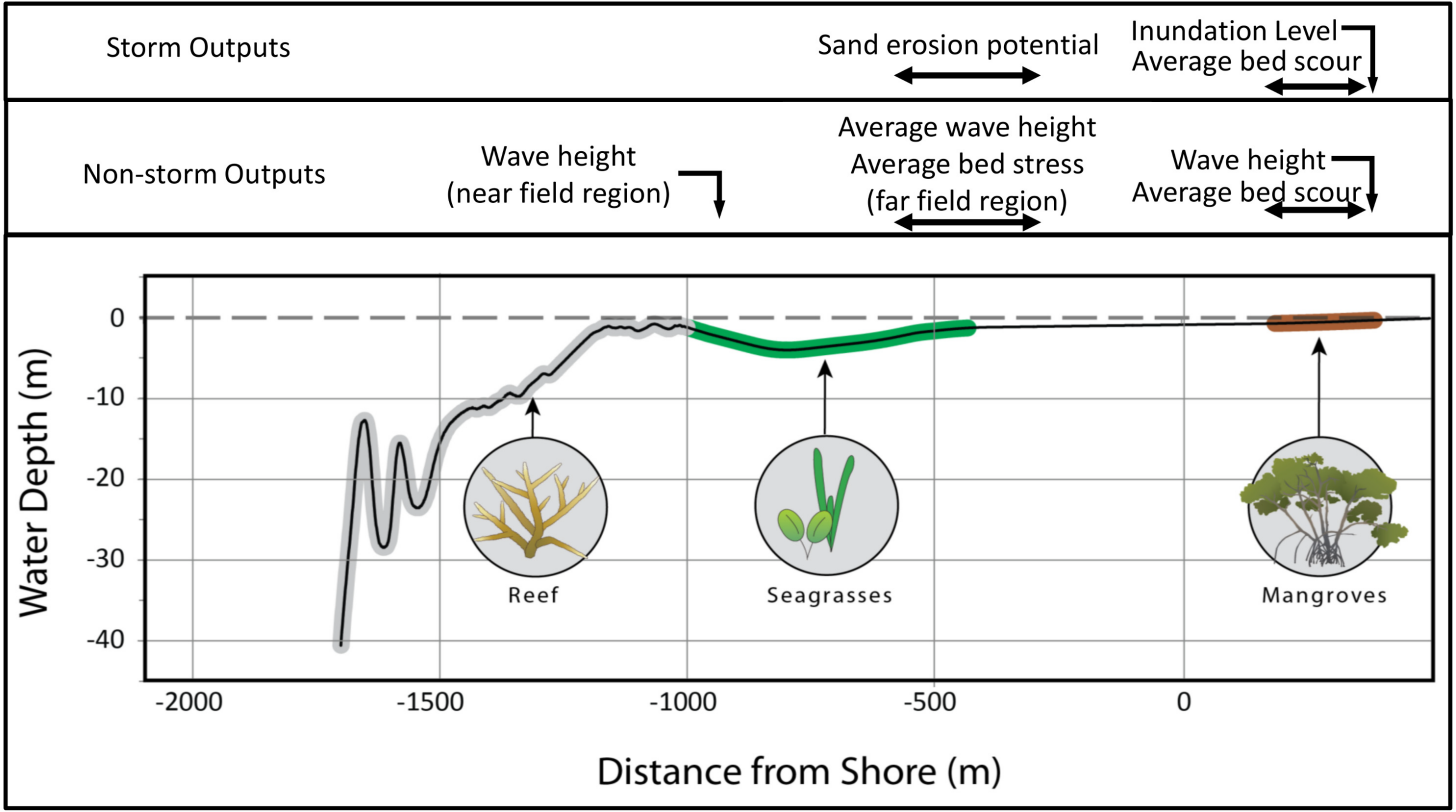
Locations of output metrics computation. Metrics are computed during non-storm and storm conditions at similar locations in both profiles. Locations are approximate.

During hurricanes, high waves and surges can erode shorelines, flood coastal regions and damage structures. During non-storm periods, currents generated by wind-waves and swells can also erode shorelines, especially unvegetated muddy shorelines [73–75], and can prevent mangroves from reproducing [76,77]. Here, we quantify the relative importance of coral reefs, seagrasses and mangroves at moderating the impacts of waves and storm surge by computing a set of protection metrics along each of the profiles. The protection metrics differ for non-storm and storm conditions, but together they include changes in wave height, bed shear stress, mud bed scour volume, inundation level and sand erosion potential in the presence or absence of one of more habitats. These erosion and/or inundation metrics are useful for understanding how habitats protect coastal regions from loss of land and damages to property. Nonetheless, these simple metrics do not appropriately describe or reveal patterns in the long-term morphodynamic evolution of the Belizean coastline. Fig 3 indicates the cross-shore locations where the different metrics are computed.

To quantify coastal protection metrics during non-storm periods, we model the evolution of each wave obtained from the WW3 record over the reefs, seagrass meadows and inside the submerged mangrove forests. We examine the protection services supplied by those habitats starting at the reef, where we compute the transmitted wave heights into the lagoon for different coral health conditions. We then compute the evolution of those transmitted waves over the seagrass meadows, assuming those plants are present or absent. We also gage whether seagrasses and coral reefs increase the long-term protection of coastal environments by computing in the nearshore region–the region extending from -0.5 m, the offshore edge of the mud flat and seaward of the mangrove forest, to 10 m inside the seagrass bed–average values of wave height and bed shear stress *τ*_*b*_. Values of *τ*_*b*_ are also compared to a typical value of critical stress threshold for sand motion *τ*_*c*_ = 0.16 N/m^2^ [78] to estimate the waves’ potential to mobilize and transport sediment. Finally, we estimate the relative role of corals, seagrasses and mangroves at protecting shorelines by computing the volume of mud bed scoured in the submerged mangrove forest [51], and the wave height at the landward edge of the submerged forest, in the presence or absence of one or more of those habitats.

During the hurricane, we compute the evolution of the offshore wave in the presence and absence of one or more habitats, assuming that waves travel over the computed storm surge. We quantify the protective services supplied by the different combinations of habitats by comparing the volume of mud bed scoured from the submerged mud bed. We also compare inundation levels at the landward limit of the mud bed, where inundation levels are defined as the sum of surge elevation, wave height and wave-induced mean water level, or setup, $\bar{\eta }$ [51]. We estimate the setup following Guannel et al. [51]:

$$\rho g\left(h+\bar{\eta }\right)\frac{\partial \bar{\eta }}{\partial x}+\frac{\partial S_{xx}}{\partial x}+\rho \frac{gk}{16\tanh kh\sqrt{\pi }}\left(C_{dr}d_{r}N_{r}\alpha_{r}+C_{dt}d_{t}N_{t}\alpha_{t}\right)H^{3}=0$$ (8)

where *S*_*xx*_ is the radiation stress caused by waves and rollers [79,80], and *α*_*r*,*t*_ = *h*_*r*,*t*_ / *h* is the relative height of plant’s roots and trunk. We assume $\bar{\eta }\equiv 0$ at the back reef, ignoring reef circulation [72]. Lastly, we compare the ability of the various combinations of habitats to mobilize and transport sand in the nearshore region, offshore of mangroves, by computing values of the average sand erosion potential [81], expressed in W/m:

$$I=EC_{g}\frac{U_{Off}}{u_{b}}$$ (9)

where *U*_*off*_ is the depth-average undertow along the profile [82].

## Results

We first examine how coral reefs, seagrasses and mangroves protect coastal regions against wind-waves and swells during non-storm conditions. Next, we examine how those habitats protect coastal regions during a hurricane (Table 1).

### Services Delivered during Non-Storm Periods

To quantify the relative protective benefits of the three types of habitats, we first quantify the protection services supplied by coral reefs alone. Next we quantify the combined and individual effect of live reef and seagrass on waves and bed stress nearshore. Finally, we quantify the combined and individual effect of live reef, seagrass and mangroves on waves at the shoreward edge of the submerged mangrove forest and on the volume of mud bed scoured from the submerged forest floor.

### Protection Supplied by Coral Reefs in Non-Storm Periods

We used the same measured reef profile in both the fringing and barrier reef systems (see Section 2.1). Consequently, wave heights transmitted by the reefs and observed immediately shoreward of the reef flats are also identical in the two systems. Therefore, results presented in this section apply to both systems.

We find that reefs are natural breakwaters [21], but their effectiveness varies with their level of coral cover. In the present day scenario, in the absence of coral cover on reefs (“Bare Reef”, Fig 2), offshore maximum and mean wave heights are reduced in the lagoons to 9% and 21% (0.31 and 0.28 m) of their initial values (Fig 2, Table 3). Corals on the reefs further dissipate incoming wave energy: a dead reef transmits only 6% and 17% (0.24 and 0.23 m) of the incoming maximum and mean offshore wave heights, and a live reef further reduces these two waves to 0.4% and 12% (0.18 and 0.16 m) of their initial offshore values.

**Table 3:** Summary of non-storm wave climate statistics in the lagoon for different reef conditions.

	Ocean Waves	Bare Reef	Dead Coral Present	Live Coral Present and Future	Dead Coral Future
Mean Wave Height [m]	1.32	0.28	0.23	0.16	0.81
Maximum Wave Height [m]	3.80	0.31	0.24	0.18	0.90

**Fig 4:**
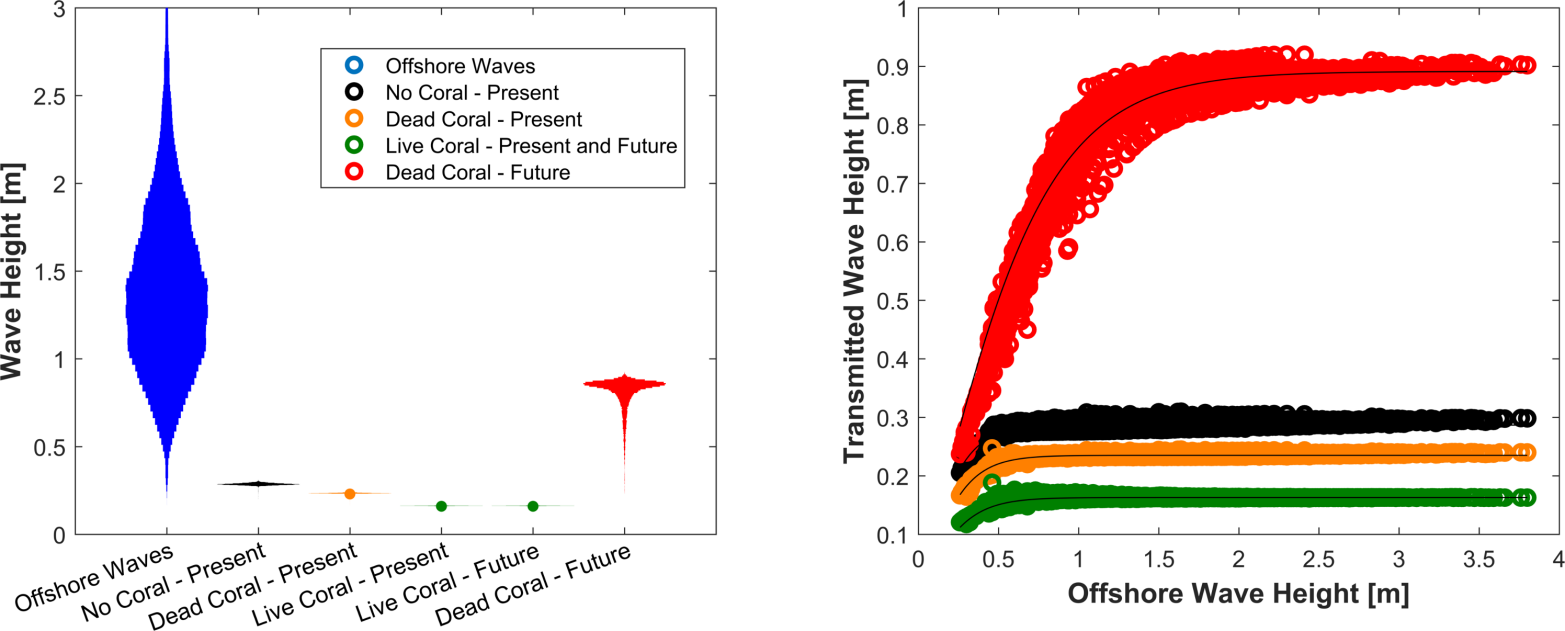
Wave distribution in the lagoon for different reef health conditions. “No Coral–Present” data were computed assuming a smooth and dead reef. Note that we represented the distribution of waves for live and dead corals under present conditions by dots in order to improve readability.

As mean sea level increases, live reefs maintain their relative depth and coral cover, while dead reefs degrade further and do not grow. Consequently, live reefs provide the same amount of wave attenuation in present and future scenarios (Fig 4, Table 3). In contrast, dead reefs under present conditions become bare and smooth in the future, and the height of the transmitted maximum and mean offshore wave heights increase fivefold to 0.90 and 0.81 m, respectively.

These results suggest that reefs act as dynamic low-pass filters for waves [21,83] (Fig 4), where the transmitted wave height *H*_*t*_ can be computed as *H*_*t*_ = *A*_1_ tanh [*H*_*o*_ / *A*_2_] (*R*^2^ ≈ 0.6 for the “Bare Reef” scenario, black circles Fig 3B, *R*^2^ > 0.8 for all other scenarios). The presence and condition of corals on the reef, or the reef’s health, control the values of the maximum transmitted wave height, *A*_1_, and of the minimum offshore wave height filtered by the reef, *A*_2_.

**Fig 5:**
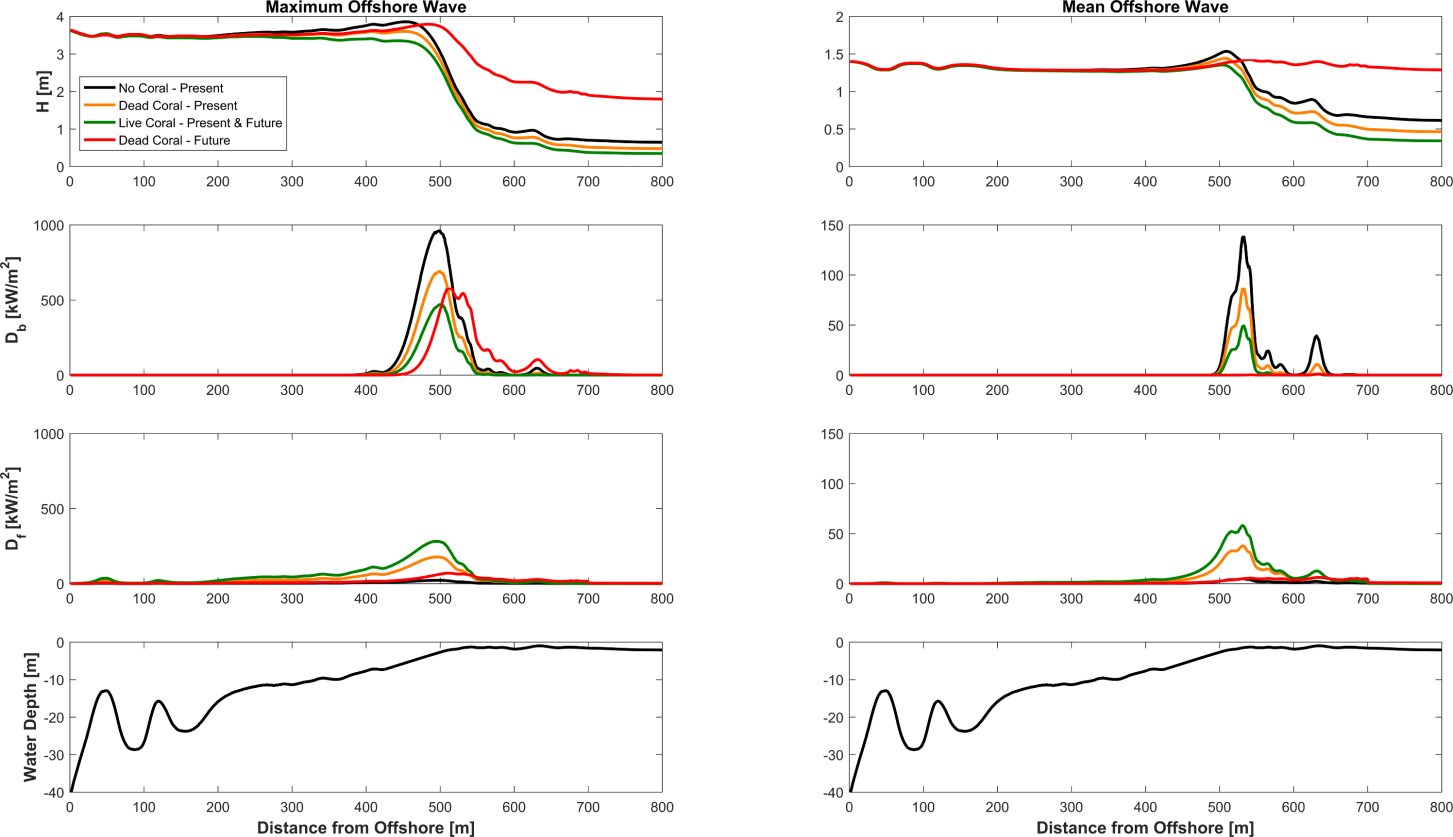
Relative importance of breaking and frictional dissipation over the coral reef. Profiles of wave height (top subplot), breaking dissipation (second subplot), frictional dissipation (third subplot) over the coral reef profile (bottom subplot), for the maximum offshore wave height (left) and mean wave height (right), for different combination of coral presence and absence. “No Coral–Present” data were computed assuming a smooth and dead reef.

Analysis of energy flux dissipation due to breaking and bottom friction indicates that, in the absence of corals, nearly all of the incoming wave energy is dissipated by breaking on the reef face and rim (Fig 5). In the presence of corals, waves still dissipate their energy via breaking, but the importance of frictional dissipation increases, as previously observed by Lowe et al. [63]. When corals on the reef die, frictional dissipation is only slightly smaller than breaking dissipation; when the corals are alive, frictional dissipation is nearly equal to breaking dissipation for large waves, and it can even exceed breaking dissipation for smaller waves (see also S2 Fig.).

**Fig 6:**
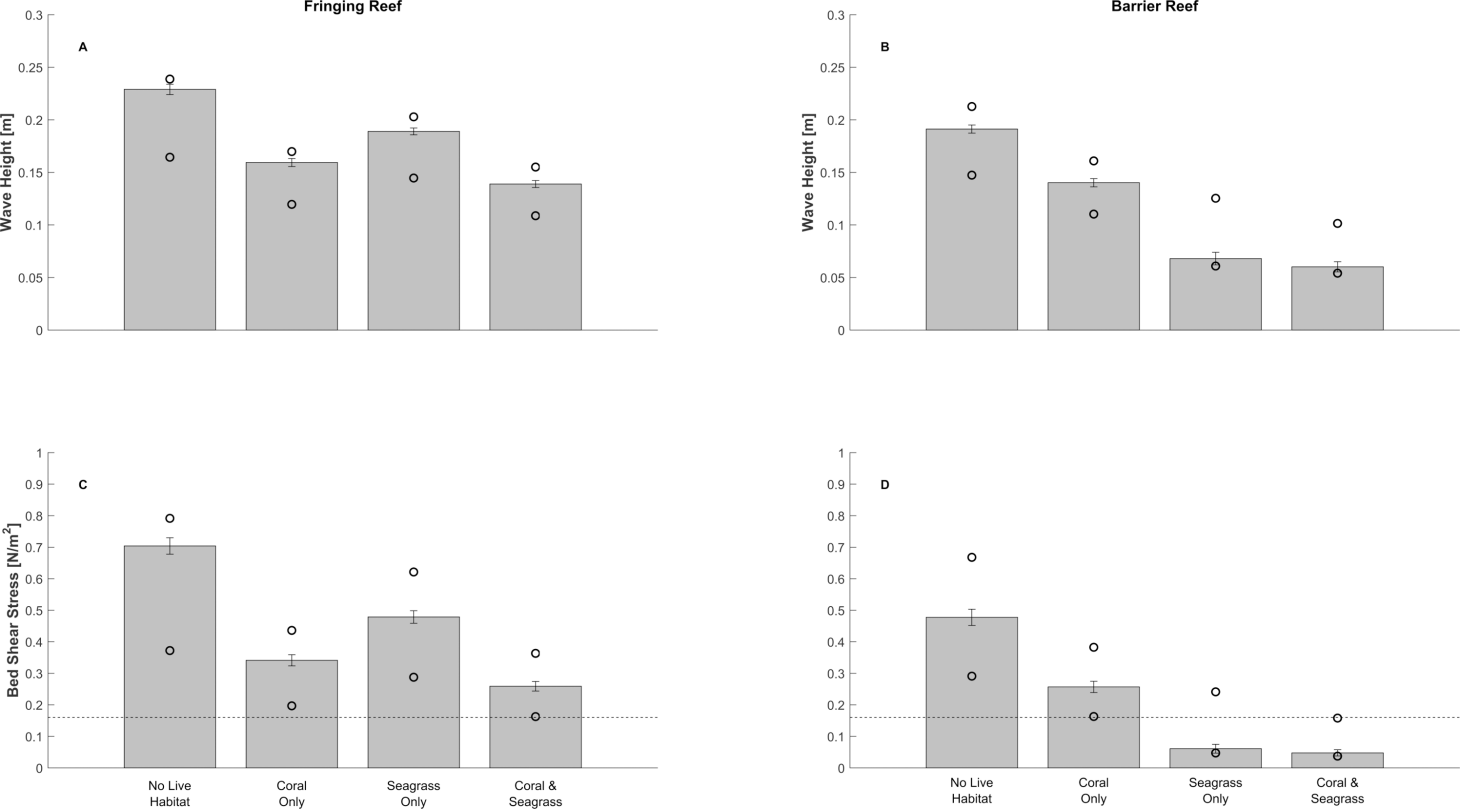
Protective role of corals and seagrasses during non-storm conditions under present conditions. Bar plots of average values of wave height (top subplots) and bed shear stress (bottom subplots) computed for different combinations of live reef and seagrass meadows present under present sea-level conditions. The dashed horizontal line in the bottom subplot represents the critical shear stress for sand motion. Vertical tick marks indicate 1 standard deviation value around the mean. Circles represent minimum and maximum values. See S3 Fig for box plot version of this figure and analogous results for future sea-level rise scenario.

Live corals on the reefs also influence the values of the protection metrics nearshore, or the far field region (Figs 3 & 6). Under present day sea-level conditions, if the corals on the barrier reef are dead, nearshore waves are 0.19 m on average, or 35% higher than nearshore wave height values obtained if corals on the reef were alive. A dead barrier reef also causes average nearshore bed shear stresses to be 85% greater, at 0.48 N/m^2^, than what would have been observed if the reef were alive (Fig 6). And as sea level rises, the far field impacts of coral reef degradation are even more pronounced [83]: nearshore waves and bed shear stresses in a future dead and bare barrier reef profile are, on average, respectively 3.5 and 13 times higher than what would have been observed if the reef were still alive.

We find similar results for the fringing reef profile. Under present day conditions, nearshore waves and bed shear stresses shoreward of a dead fringing reef are, at 0.23 m and 0.70 N/m^2^, respectively, 50% and 100% greater than values obtained for a live reef. And under future sea-level conditions, nearshore wave height and bed shear stress shoreward of a dead and bare reef are 4 and 17 times greater, respectively, than the values obtained for a live reef. Note that, nearshore wave height and bed shear stress values are lower in the barrier reef than in the fringing reef, since waves in the longer lagoon have more time (space) to dissipate their energy via bottom friction. Also, in both profiles, dissipation occurs via bottom friction; waves barely break in the lagoons.

#### Protection Supplied by Coral Reefs and Seagrasses in Non-Storm Conditions

In both the barrier and fringing reef profiles, once waves pass the reef, they encounter and propagate through seagrass meadows that further attenuate the incoming waves, resulting in lower wave heights and bed stresses nearshore than the no live habitat and the coral only scenarios (Fig 6).

In the barrier reef lagoon, seagrass meadows reduce average non-storm waves in the nearshore to 0.07 m, or half the mean wave height of the coral only scenario (note that most of the protection supplied by seagrasses comes from the nearshore meadow; the offshore meadow matters less–S4 Fig). Seagrasses also reduce bed shear stress by more than 70% to 0.05 N/m^2^, which is below the threshold for sediment motion (Fig 6B and 6D, “Coral & Seagrass” vs. “Coral Only”). Even without live corals on either reef, seagrasses still reduce waves and bed shear stresses nearshore by more than 60% to 0.07 m and 0.06 N/m^2^, compared to 0.19 m and 0.48 N/m^2^ with a dead reef and no seagrass (Fig 6B and 6D, “Seagrass Only” vs. “No Live Habitat”). These results indicate that together, live corals and seagrasses provide more protection benefits in the barrier reef profile than either of those habitats alone. These results also suggest that seagrasses moderate the far field consequences of reef degradation at the coastline. We observe an analogous pattern in the sea-level rise scenario (S3 Fig).

For the fringing reef profile, the combination of live coral and seagrass is also the most effective for reducing wave heights and bed shear stress. When operating together, they reduce nearshore wave height by nearly 40% (0.13 m vs. 0.22 m, Fig 6A) and bed shear stress by more than 60% (0.26 N/m^2^ vs. 0.7 N/m^2^, Fig 6C). Individually, both habitats still help reduce nearshore wave height and bed stresses, but their relative importance is reversed compared to the barrier reef profile, mostly because the lagoon is shorter and the reef closer to shore. In the absence of live corals, seagrasses alone reduce nearshore wave height by 13% to 0.19 m and bed shear stress by 30% to 0.48 N/m^2^, compared to what we observe without any live habitat (Fig 6A and 6C, “Seagrass Only” vs. “No Live Habitat”). Corals alone, on the other hand, reduce wave heights by 27% to 0.15 m, and bed shear stress by 51% to 0.37 N/m^2^ (Fig 6A and 6C, “Coral Only” vs. “No Live Habitat”). Consequently, corals on the fringing reef are more effective at moderating the nearshore wave climate than seagrasses, although seagrasses offset the loss of live corals to some extent. Similar patterns are observed in the sea-level rise scenario, except that habitats, together or individually, reduce nearshore wave height and bed stress by more than 400%, on average (S3 Fig).

Interestingly, regardless of the presence of habitats, nearshore wave height and bed shear stress values in the fringing reef profile are higher than in the barrier reef profile. This difference is due, among other bathymetric variations, to the fact that the fringing reef lagoon is shorter than the barrier reef lagoon, so waves do not dissipate as much of their energy through bottom friction. Consequently, coral and seagrasses help reduce bed stress nearshore in the fringing reef profile, but not below the threshold of motion, as observed in the longer reef profile.

### Protection Supplied by Coral Reefs, Seagrasses and Mangroves in Non-Storm Conditions

**Fig 7:**
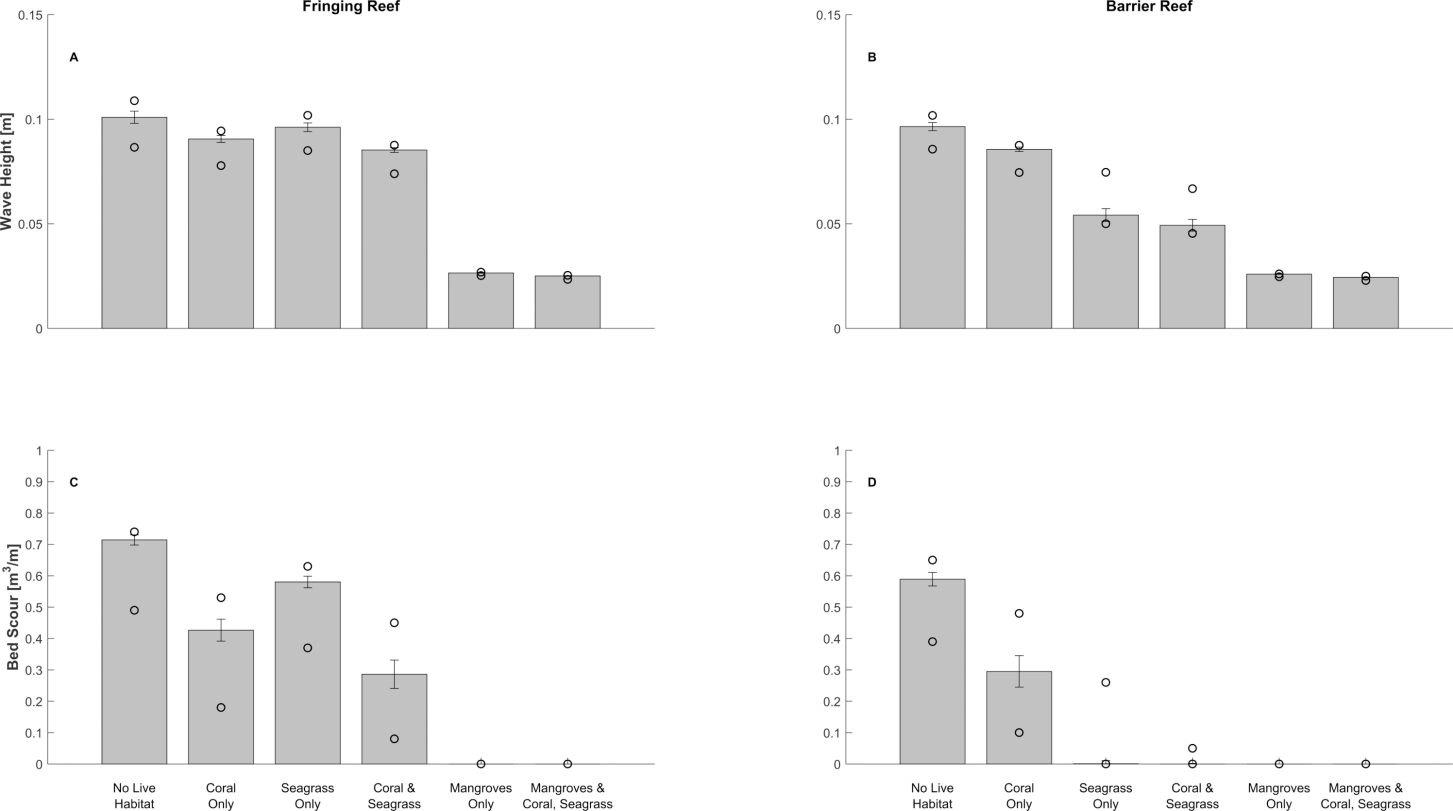
Protective role of corals, seagrasses and mangroves during non-storm conditions under present sea-level conditions. Bar plot of average wave height at the shoreward edge of the submerged mangrove forest (top subplots) and bed scour volume over the submerged mangrove forest (bottom subplots) computed for different combinations of live reef, seagrass meadows and mangroves presence, under present sea-level conditions. Vertical tick marks indicate 1 standard deviation value around the mean. Circles represent minimum and maximum values. See S5 Fig for box plot version of this figure for a future sea-level rise scenario.

After waves have travelled over the coral reef and seagrass meadows, they encounter the mangrove forests. In both reef profiles, mangroves attenuate all non-storm waves by approximately 70% of the nearshore wave height, to only 2–3 cm, regardless of the presence of corals or seagrass offshore (Fig 7A and 7B, “Mangroves Only” and “Mangroves & Coral, Seagrass”). Mangroves alone also eliminate any potential for erosion of the forest’s floor during non-storm conditions (Fig 7C and 7D, “Mangroves Only” and “Mangroves & Coral, Seagrass”).

The effectiveness of mangroves can be partially attributed to the fact that waves entering the forest are relatively small, having previously travelled over a shallow, mildly sloping bed. But this effectiveness is still mostly due to the frictional drag that their dense trunks and roots induce on the water column. Thus, as observed in other locations, mangroves alone are very effective at preventing soil loss and shoreline erosion [73,75].

Despite the ability of mangroves to completely attenuate all waves, we find that corals and seagrasses can still play an important role. In the barrier reef profile, in the absence of any live habitat, nearshore waves at the shoreward edge of the submerged forest are, on average, 0.1 m high (Fig 7B, “No Live Habitat”), which results in approximately 0.6 m^3^/m of mud scoured from the submerged forest (Fig 7D, “No Live Habitat”). In the presence of live corals and seagrasses, average wave height at the shoreward edge of the submerged forest and mud scour volumes in the forest decrease to 0.05 m and 0.0 m^3^/m (Fig 7B and 7D, “Coral and Seagrass”). Interestingly, if the reef dies but seagrasses remain present, wave heights only increase by 1 cm, and no bed scour occurs (Fig 7B and 7D, “Seagrass Only”). However, if the reef is alive and seagrasses are absent, those quantities increase to 0.09 m and 0.3 m^3^/m (Fig 7A and 7C, “Coral Only”). Consequently, in the barrier reef profile, seagrasses provide more protection benefits than coral reefs when mangroves are absent, and, once again, seagrasses can at least partially compensate for the degradation of the reef. Values increase slightly in sea-level rise scenario, but the pattern remains the same (S5 Fig).

Results differ for the fringing reef lagoon. In the absence of any habitat, non-storm nearshore waves are, on average, 0.1 m high at the shoreward edge of the submerged forest, and 0.7 m^3^/m of the muddy forest floor is scoured (Fig 7A and 7C, “No Live Habitat”). However, if the reef dies but seagrasses are still present, wave heights and the volume of mud bed scoured barely change (0.1 and 0.6 m^3^/m, respectively, Fig 7A and 7C “Seagrass Only”). In contrast, if the seagrass disappears but the reef is alive, those quantities decrease to 0.09 and 0.4 m^3^/m, respectively (Fig 7A and 7C “Coral Only”). Thus, compared to the results for the barrier reef, live corals provide more protection services than seagrasses in the fringing reef profile, and seagrasses do not compensate for coral reef degradation.

Overall, we find again that multiple habitats provide more benefits than any single habitat alone: in the presence of a live reef and seagrasses, the average wave height at the shoreward edge of the submerged forest and the volume of mud scoured in the forest decrease to 0.08 m and 0.3 m^3^/m, respectively (Fig 7A and 7C “Coral & Seagrass”). Values increase slightly in the sea-level rise scenario, but the pattern remains the same (S5 Fig).

### Services Delivered during Storm Conditions

We quantify the combined and individual coastal protection benefits supplied by live corals, seagrasses and mangroves during a typical hurricane. We measure their ability to reduce inundation levels (including surge height, wave height and wave setup) shoreward of the mangrove forest, and to reduce mud bed scour in the mangrove forest. We also quantify the combined and individual importance of live corals and seagrasses at reducing the potential for sand erosion just offshore of the mangrove forest.

**Fig 8:**
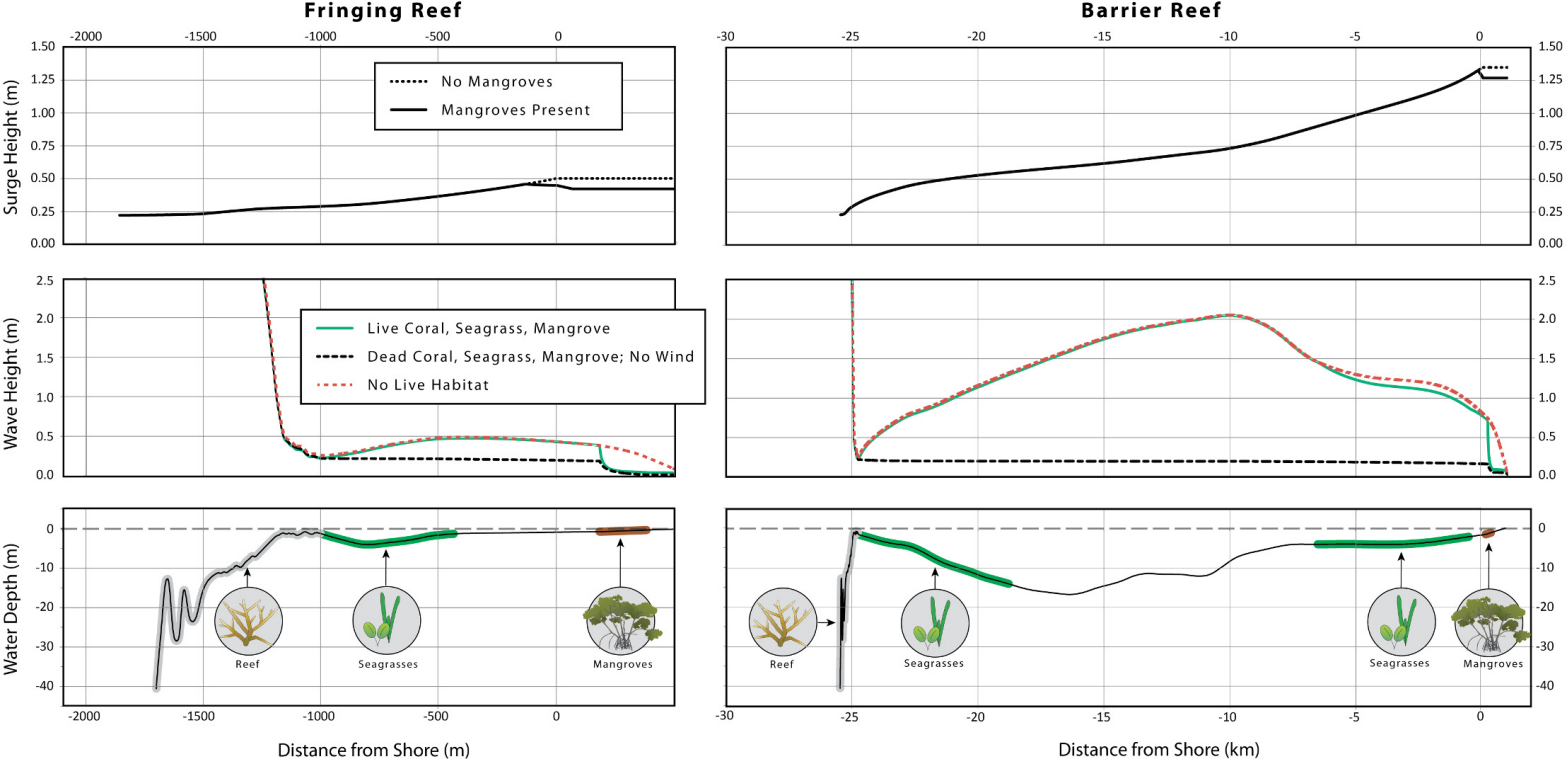
Moderating effects of natural habitats on storm surge and waves. Profiles of surge (top subplots), wave height (middle subplots) and bathymetry, with habitats (bottom subplot) in the fringing and barrier reef profiles during the synthetic hurricane. Profiles of wave height in the absence of wind are shown to illustrate the extent of wave re-generation that occurs in the lagoons.

**Fig 9:**
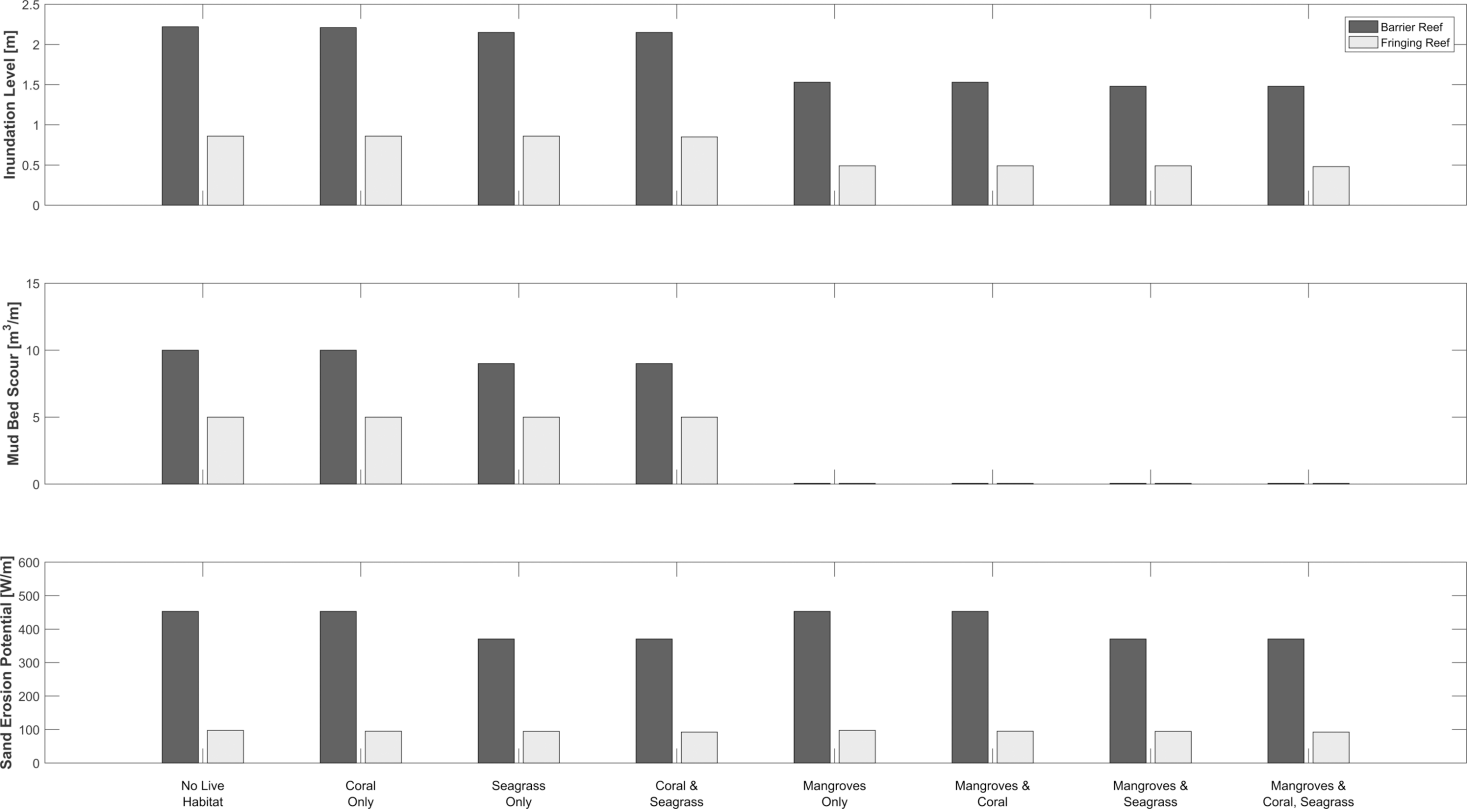
Protective role of corals, seagrasses and mangroves during storm conditions. Coastal protection services provided by coral reefs, seagrass beds and mangroves, under present day sea-level conditions, for various combinations of live and dead habitats during a hurricane. Patterns are the same for the future sea-level rise scenario (S6 Fig).

We find that, in the absence of habitats, differences in the geomorphology of the two profiles yield significant differences in inundation and sediment loss. Because the barrier reef lagoon is so long, the hurricane generates a 1.2 m surge in the barrier reef profile compared to only a 0.5 m surge in the fringing reef profile. Also, after breaking over the reefs, hurricane waves re-generate to a maximum height of 2.1 m in the barrier reef lagoon compared to only 0.5 m in the fringing reef lagoon (Fig 8). Therefore, in the absence of habitats, inundation levels, mud bed scour volumes, and sand transport potential values are more than 60% lower in the fringing reef profile than in the barrier profile (Figs 8 & 9): once again, the distance of the reef from the shoreline has profound repercussions on the lagoon hydrodynamics and sediment transport patterns [19,83].

Examining the role of the different habitats, we find that in both profiles mangroves alone systematically reduce inundation levels and mud bed scour volumes, while live corals and seagrasses play a relatively minor role (Figs 8 & 9 all combinations containing mangroves). Mangroves lower the surge at the landward edge of the mud bed by 10 cm in the barrier reef, and 8 cm in the fringing reef; they reduce wave heights by more than 70% (66 cm to 15cm, in the barrier reef, and 28 cm to 8 cm in the fringing reef); and they also decrease wave setup by more than 70% (21 cm to 6 cm in the barrier reef, and 8 cm to a slight setdown in the fringing reef). Thus, for both reef profiles, mangroves alone decrease the inundation level at the landward edge of the mud bed by more than 35% (2.22 m down to 1.48 m in the barrier reef, and 0.86 m to 0.48 m in the fringing reef). The effectiveness of the mangroves can be partially, but not entirely, explained by the fact that they are the only habitat in this study that is able to moderate storm surge [18]. However, we find that most of their effectiveness is due to the strong moderating effect of mangroves on waves: after re-computing outputs for the storm scenario ignoring storm surge reduction due to mangroves, we found similar results to those presented in Fig 9 (S7 & S8 Figs).

Besides mangroves, we find that live corals and seagrasses play minor but different roles in the two profiles. In the barrier reef system, live and dead corals have little influence on wave height and other coastal protection metrics (Fig 9, all combinations that do not contain mangroves): waves have the time and space to re-generate in the deep and long lagoon as they approach the shoreline, eliminating any far field effect of the presence of corals. Seagrasses, on the other hand, decrease the wave height by 1.5 cm and setup by 5 cm at the landward edge of the mud bed, and reduce the volume of mud scoured from the forest floor by 1 m^3^/m (Fig 9, “No Live Habitat”, “Seagrass Only” and “Coral and Seagrass”). This surprising (albeit minor) far-field effect of the seagrass meadows can be explained by the cumulative impact of the small but continuous force those plants exert on waves. In the fringing reef however, neither live corals on the reef nor seagrasses in the short lagoon affect inundation or bed scour (Fig 9, all combinations that do not contain mangroves).

The small and contrasting role played by seagrasses and corals is further demonstrated by the results for sand erosion potential computed just offshore of the mangroves (Fig 9). In the barrier reef, seagrasses decrease the sand erosion potential by roughly 20% (Fig 9, “No Live Habitat”, “Seagrass Only” and “Coral and Seagrass”); the presence of corals on the reef matters little. A similar pattern is observed in the sea-level rise scenario (S6 Fig).

Finally, in the fringing reef, corals and seagrasses have a negligible influence on sand erosion potential presently (roughly 3% reduction; Fig 8, “No Live Habitat”, “Seagrass Only” and “Coral and Seagrass”). In the future sea-level rise scenario, however, a dead and bare reef causes the sand erosion potential to increase by 30% relative to present day conditions (S6 Fig).

## Discussion and Conclusion

In this paper, we investigate the contribution of coral reefs, seagrass meadows and mangroves to the protection of coastal areas in two idealized reef systems (i.e., barrier and fringing reef), under present day and future (+1 m) sea-level conditions, and during both non-storm and storm conditions. We find that together, these habitats substantially moderate incoming wave energy, inundation levels and loss of mud sediment [84,18,51,85], and ultimately protect the coast better than any one habitat alone. More precisely, we find that while mangroves alone can deliver most of the above-mentioned coastal protection benefits, corals and seagrasses also moderate the nearshore wave climate. Thus, corals and seagrasses are likely to help reduce the risk of shoreline erosion; promote shoreline stability offshore of the mangroves [9,20,51,86]; reduce nearshore currents, which could allow mangroves to recruit and maintain a viable population [29,76,87]; and ultimately increase the resilience of coastal regions against hazards [9,86]. In other words, even though mangroves fill an entirely different coastal protection “niche” than corals and seagrass, the latter habitats are interchangeable for a subset of important coastal protection functions.

Another major finding is that the ability of different habitats to provide coastal protection services varies as a function of local geomorphology, or setting, and forcing conditions [88,89]. During non-storm conditions, habitats in the long and deep barrier reef profile reduce more wave energy than in the short and shallow fringing reef. Conversely, surge and maximum wave height during hurricanes are larger in the barrier reef profile than in the fringing reef profile. As a consequence, habitats in the barrier reef profile cannot protect coastal regions to the same degree as habitats in the fringing reef profile (Fig 9). Additionally, these differences in setting also yield differences in habitat performance. In the fringing reef, corals on the reef supply more protective services than the seagrasses during both storm and non-storm conditions, especially if the reef can keep up with sea-level rise. On the other hand, in the barrier reef, nearshore seagrasses supply more protection services than live corals, and under certain circumstances, seagrasses can compensate for the impacts of short-or long-term degradation of the reef. These differences in the nature and location of the main supplier of protection services has rarely been highlighted in the nearshore processes literature and might warrant further investigation, especially if some habitats are expected to be negatively affected by the impacts of climate change.

Our results also illustrate the importance of taking into account the presence of live corals when computing the ecosystem service function of reefs [19,42,83], something that has been overlooked in previous studies [20,21,90]. Live corals provide additional wave dissipation benefits, and ensure the long term provisioning of services by the reef by growing and accreting with sea level rise [83,91,92]; in the absence of corals, reefs are mostly bathymetric perturbations.

Finally, results presented herein also illustrate the importance of clearly identifying the metrics used to quantify protection services. Live corals in the two profiles supply the same level of protection if the service (e.g., wave height attenuation) is measured at the shoreward edge of the reef. However, if the service is measured nearshore, live corals in the fringing and barrier reef profiles do not provide the same amount of services. The same argument can be made for seagrass meadows far from the coast (S3 Fig). This point is critical to properly estimating coastal protection metrics that matter to people.

In conclusion, we demonstrate the importance of taking an integrated approach to assessing the protection services supplied by marine habitats when crafting conservation or restoration plans. The merits of such an integrated approach has been established for fisheries management [93,94]. Our results reinforce the importance of such an approach by showing that the successful design of nature-based coastal protection schemes also requires quantifying and understanding the interaction, or the hydrodynamic connectivity, between biotic and abiotic features that characterize a marine seascape. Such an approach would account for the varied ways in which different habitats reduce risks from coastal hazards and thus improve our ability to manage marine seascapes to protect communities in the future.

## Supporting Information

S1 Fig Non-storm wave record. Left: Complete wave record from WW3 grid point. Center: Distribution of wave height and wave period. Right: Wave rose of the complete record. Wave rose of non-storm record is similar.
(EPS)

S2 Fig Relative importance of breaking and frictional dissipation for all non-storm waves travelling over the reef. Left: Average ratio of breaking and frictional dissipation over the reef profiles. Center: scatter plot of breaking dissipation; solid line represents an approximation of the best fit (R^2^>0.8). Right: scatter plot of frictional dissipation; solid line represents an approximation of the best fit (R^2^>0.8). Results presented for different combinations of coral presence and absence, under present and future conditions.
(EPS)

S3 Fig Protective role of corals and seagrasses during non-storm conditions in the future sea-level scenario. Box plots of wave height (top subplots) and bed shear stress (bottom subplots) values computed as a function of the presence or absence of live coral or seagrasses. Circles represent mean values.
(EPS)

S4 Fig Role of seagrass meadow location in coastal protection. Box plots of wave height values computed in both profiles in the presence and absence of a portion or the whole meadow. The offshore meadow is the one closest to the reef. The nearshore meadow is the one closest to the shore. In the fringing reef, the seagrass meadow was split in two equidistant meadows. Circles represent mean values.
(EPS)

S5 Fig Protective role of corals, seagrasses and mangroves during non-storm conditions in future sea-level conditions. Box plots of wave height (top subplots) and bed scour (bottom subplots) values computed as a function of different combination of presence or absence of live coral, seagrasses and mangroves. Circles represent mean values.
(EPS)

S6 Fig Coastal protection services provided by coral reefs, seagrass beds and mangroves, in future sea-level conditions, for various combinations of live and dead habitats during a hurricane. (EPS)

S7 Fig Coastal protection services provided by coral reefs, seagrass beds and mangroves, in present day conditions, for various combinations of live and dead habitats during a hurricane. Surge attenuation by mangroves is ignored.
(EPS)

S8 Fig Coastal protection services provided by coral reefs, seagrass beds and mangroves, in future sea-level conditions, for various combinations of live and dead habitats during a hurricane. Surge attenuation by mangroves is ignored.
(EPS)
